# Serum level of vitamin D and trace elements in children with recurrent wheezing: a cross-sectional study

**DOI:** 10.1186/1471-2431-14-270

**Published:** 2014-10-16

**Authors:** Metin Uysalol, Ezgi Pasli Uysalol, Yasin Yilmaz, Gunes Parlakgul, Tulin Ayse Ozden, Hayriye Vehid Ertem, Beyhan Omer, Nedret Uzel

**Affiliations:** Department of Pediatrics, Istanbul Faculty of Medicine, Istanbul University, Capa, 34390 Istanbul, Turkey; Department of Pediatrics, Institute of Child Health and Istanbul Faculty of Medicine, Istanbul University, Trace Element Unit, Capa, 34390 Istanbul, Turkey; Institute of Child Health, Istanbul University, Capa, 34390 Istanbul, Turkey; Department of Biochemistry, Istanbul Faculty of Medicine, Istanbul University, Capa, 34390 Istanbul, Turkey

## Abstract

**Background:**

We aimed to show the relationship between recurrence of wheezing and serum levels of vitamin D, zinc, and copper in wheezy children compared with a healthy group.

**Methods:**

In this cross sectional study, seventy-three children with wheezing and seventy-five controls were included without a follow-up period. The clinical characteristics of the children were assessed, the asthma predictive index and temporal pattern of wheeze were determined. The serum levels of vitamin D, zinc, and copper were measured. Pearson correlation analysis was used to evaluate the relationship between homogeneously distributed variables.

**Results:**

Thirty-two of the seventy-three children (43.8%) had more than three wheezing attacks (recurrent wheezing). The Asthma Predictive Index index was positive in 26 patients (35.6%). When classified to temporal pattern of wheeze, fifty-three of the study group (72.6%) had episodic wheezing and the remainder (27.4%) was classified as multiple-trigger wheezing. We found no overall significant difference between the study and control group in terms of vitamin D and trace elements . The vitamin D and zinc levels were significantly lower and serum copper and copper/zinc ratio was significantly higher in patients with recurrent wheezing (p =0.03, p <0.01, p =0.013, p <0.01, respectively) positive Asthma Predictive Index and multiple-trigger temporal pattern of wheeze compared with patients with non- recurrent wheezing, negative Asthma Predictive Index and episodic temporal pattern of wheeze.

**Conclusion:**

It may be postulated that for the determination of asthma risk in patients with recurrent wheezing, the serum level of vitamin D, copper and zinc can be used as a routine biomarker alongside the Asthma Predictive Index and temporal pattern of wheeze.

## Background

Wheezing is very common complaint on admission to the pediatric emergency department and is one of the important causes of mortality and morbidity worldwide [1]. Thirty percent of children have at least one wheezing attack before the age of three, and fifty percent of children have before the age of six. It has been reported that recurrent wheezing attacks might frequently be seen before school-age and forty percent of children with recurrent wheezing attacks can suffer from wheezing symptoms in their later life [2]. It has also been shown that early wheezing attacks may be a first sign for ensuing asthma [3], and that eighty percent of patients with asthma had wheezing symptom in their first year of life [4]. In a study from Turkey consisting of 46,813 children, wheezing prevalence was 15.1% [5].

The relationship between wheezing in infancy and ensuing development of asthma has been under investigation for a long time. There is also detailed research determining the risk factors of recurrent wheezing. It is thought that genetic and individual immunologic features along with environmental factors might be responsible for childhood temporal pattern of wheeze [6]. Some kind of indexes and laboratory findings can be used to determine in which children wheezing will recur or develop into asthma. For the ensuing asthma risk, the Asthma Predictive Index (API) (frequency of wheezing, asthma history in parents and atopy history) can be performed in clinical settings [7].

The European Respiratory Society (ERS) has classified temporal patterns of wheeze according to cause of wheezing and clinical characteristics as viral (episodic) wheeze (EW) and multiple-trigger wheeze (MTW). However, the differential of temporal pattern of wheeze in infancy is difficult. Most studies about wheezing in infancy have shown that there are other causes related to respiratory tract infections with multifactorial etiology in pathogenesis of wheezing [8].

Temporal pattern of wheeze with positive API can affect developing asthma [4, 9, 10]. Another study has shown that a positive API and MTW or a severe attack can increase the risk of asthma at school age [11].

Recently, it has been reported that the frequency of asthma has increased. Some theories have been postulated for this reason: changing environmental factors, western life-style, and eating habits [12]. The effect of vitamin D (VD) and trace elements (TE) on asthma has been discussed in some studies. Nutrition including foods containing vitamin D and trace elements might be effective in recurrent wheezing (RW) and asthma. It has also been reported that there is a positive relationship between the decreased intake of antioxidants and increased incidence of asthma [13].

According to recent studies, vitamin D and trace elements can affect the host immune system in terms of anti-inflammatory and antioxidant features. Differing dietary characteristics from region to region may cause a decreased intake of vitamin D and TE, and this can result in diminished antioxidant micronutrients, in turn increasing oxidant damage and derangement of the immune system [14]. All these factors can cause airway inflammation but the certain roles of these vitamin and elements are not clear.

Trace elements exist in the structure of the antioxidant enzyme. These enzymes act as part of the immune system and can also change viral genome by regulating host immune system. It has been reported that a diet that is poor in antioxidants may render the host vulnerable to reactive oxygen species (ROS). Vitamin D and major trace elements such as zinc (Zn) and copper (Cu) have immune modulator effects and thus can affect the course of respiratory tract infections (RTIs) [15].

In this study, we aimed to reveal the relationship between recurrence and severity of wheezing and levels of serum vitamin D, zinc, and copper in wheezy children compared with a healthy group. Our second aim was to investigate the relationship between serum level of vitamin D and TE with API and temporal pattern of wheeze. Our third aim was to show whether these relationships might be used as biomarker in the follow-up and treatment of wheezing. We believe that determining additional risk factors for recurrent wheezing and asthma may guide public health policies so that more effective prevention strategies against asthma might be developed.

## Methods

### Study population

In this cross-sectional trial, the study group consisted of seventy-three patients who had been admitted to the Pediatric Emergency Department of Istanbul University, Istanbul Medical Faculty, between April, and September 2010, with a complaint of wheezing. The control group included seventy-five children who had been admitted to the emergency department with minor trauma. The control group had no history of chronic disease, lower respiratory tract disease or wheezing, and the age and sex were consistent with the study group. The whole study population resided in Istanbul.

Inclusion criteria were; aged 3 to 24 months, at least one wheezing attack, normal growth and development consistent with age, no use of systemic or inhaled steroid during or just prior to the study, no history of other disease that could be related with wheezing.

Exclusion criteria were; prematurity (less than 36th gestational week), low birth weight (less than 2500 gr), history of admission in newborn intensive care unit due to respiratory distress, history of intubation in intensive care unit, assisted ventilation during the neonatal period; patients had an underlying disease that might affect the cardiopulmonary status (e.g. congenital heart disease, symptomatic cardiac anomaly / failure, chronic lung disease, bronchopulmonary dysplasia, pulmonary tuberculosis, aspiration pneumonia, cystic fibrosis, gastro-esophageal reflux, pneumonia), immunodeficiency, neurologic or metabolic diseases, or any chronic disorder. Moreover, further exclusion criteria were; malnutrition, an inability to obtain a blood specimen after a maximum of two venipuncture attempts, and unwillingness of the parents to attend the study. In addition, we excluded patients who had been taking supplementation of vitamin D or trace elements in both groups.

Each parent was informed about wheezing and was asked whether their child had had this before, and if yes, how often. The parents were also asked to complete the questionnaire including questions about physician-diagnosed asthma, allergic rhinitis, atopic dermatitis in children and family, when these complaints started, the age of first admission, presence of risk factors for wheezing, how many wheezing attacks they had had, the number of upper RTIs and admission to emergency department in last year, consanguinity, standard of life (economic situation, home heating system, number of people per household), smoking during pregnancy, passive smoking exposure, birth information, duration of breastfeeding, and vitamin D supplement.

### Definitions

Wheezing was defined as continuance of complaints, the development of at least one wheezing attack in the previous year. The wheezy child was accepted as having more than three wheezing attacks with no known reason within the previous year [3].

The discrimination of wheezing was carried out in accordance with the advice of the European Respiratory Society Task Force [8]. Patients who had wheezing attacks only when they suffer from viral RTIs and had no complaints between attacks were accepted as episodic wheeze [8].

The Asthma Predictive Index is a valuable and reliable risk assessment index for patients who had wheezing attacks in their infancy of having asthma in their later life. The risk for the development of asthma in younger children can be estimated according to the API. According to this index, major and minor criteria are assessed when children younger than three years have more than three wheezing attacks lasting more than one day that affected night sleep. The major criteria are physician-diagnosed asthma in parents and atopic dermatitis in the child. The minor criteria are eosinophilia (>%4), wheezing without URTI and physician diagnosed allergic rhinitis in the child. The risk of having asthma in later life in children with one major criterion or two minor criteria is increased [16]. The loose index: less wheezing (<3/year) plus one major criterion or two minor criteria. The stringent index: frequent wheezing (>3/year) plus one major criterion or two minor criteria [16].

Vitamin D deficiency is defined as serum vitamin D levels of less than 20 ng/ml [10] whereas 25(OH)D of 21–29 ng/mL is considered to be insufficient and serum vitamin D levels of ≥30 ng/ml is sufficient [9].

### Physical examination

To ensure that patients evaluated by the same physician, infants with a first admission between the hours of 8 am and 5 pm were enrolled in the study. The research nurse or recruiting physician transcribed the date of enrollment, age, sex, and diagnosis from health records to the participant information form. The patients’ history was obtained and physical examination was done by a physician. Vital signs such as respiratory rate (RR), oxygen saturation, and pulse and body temperature were noted. In addition, patients’ height, weight and clinical bronchiolitis severity scores (CBSS) were obtained [17].

The severity of the infection was assessed through CBSSs of patients at admission. The baseline of the bronchiolitis clinical score was identified based on the respiratory rate, subcostal retraction, and presence of wheezing, oxygen requirement and general appearance. A clinical score less than 4 were defined as mild; 4–7 as moderate and 8–12 as severe [17].

The household person to bedroom ratio was calculated and used as an indicator of socioeconomic status. Prematurity was defined as a gestational age less than 36 weeks. Adequate breastfeeding was defined as having been breastfed for at least 6 months.

### Sample collection

Blood specimens were drawn between 8 am and 10 am, after a fasting period of at least 4 hours. Trace mineral free syringes, stainless steel needles and special trace element tubes (Becton Dickinson Vacutainer Systems) were used. The serum samples were separated after 10 minutes of centrifugation and kept in -40°C until the day of analysis. Serum zinc and copper concentrations were measured at 213.9 nm and 324.8 nm, respectively, using an atomic absorption flame emission spectrophotometer (Agilent Technologies 240Z AA, GTA-120 Graphite Tube Atomizer).

EDTA-plasma samples were used for Vitamin D determination with high-performance liquid chromatography (Spectra System AS3000, Thermo Separation Products, Thermo).

The study was approved by the Ethics Committee of Istanbul University, Istanbul Faculty of Medicine. Written informed consent was obtained from parents/guardians before enrollment.

### Statistical analysis

Vitamin D deficiency is seen in 40% of society. For cases of bronchiolitis, we assumed that vitamin D deficiency would be around 60%; and the sample size was calculated as 75 for each group with a power of 0.80 and with a confidence interval of 95%.

Statistical analysis was performed using SPSS for Windows, Release 16.0 (SPSS Inc., Chicago, IL). The investigator and the statistician were blinded to treatment allocation. The descriptive statistics (mean ± standard deviation, median, minimum and maximum values) were calculated. The data from all randomized patients were recorded on a predetermined form. The Shapiro-Wilk test was used to determine whether the data came from a normal distribution. The Kruskal Wallis H test was used if the data were not normally distributed and in case of an observation of a difference between groups, the Mann Whitney U test (with Bonferroni correction) was chosen to evaluate the difference. Student’s T test was used to compare the groups that were normally distributed with homogeneous variability. Pearson correlation analysis was used to evaluate the relationship between homogeneously distributed variables and Spearman’s rank correlation was used when the variables were not normally distributed. Categorical data such as sex were analyzed by using the chi-square test. A value of p <0.05 was considered to be statistically significant.

## Results

The study population included 148 children. The study group consisted of 73 children (mean age: 20.78 ± 3.87 months; 46 boys and 27 girls) and control group were 75 children without wheezing (mean age: 20.32 ± 3.78 months; 43 boys and 32 girls). There was no significant difference between groups regarding age and sex. The demographic characteristics and risk factors are illustrated in Table 1. The history of prenatal smoking, postnatal passive smoking exposure, kindergarten history, and history of a crowded family were significantly higher in the study group than in the control group (p <0.005).

**Table 1:** **Demographic characteristics of the study population**

	Control group	Wheezy group	
	n:75 (50.7%)	n:73 (49.3%)	p-value
	Mean ± SD	Mean ± SD	
Age (months)	18.05 ± 2.44	17.48 ± 2.36	0.149
Weight (kg)	11.89 ± 2.23	12.20 ± 2.59	0.595
Height (cm)	83.90 ± 6.51	84.08 ± 6.99	0.875
Mother Age (year)	24.71 ± 3.11	24.10 ± 3.31	0.250
Number of siblings	2.20 ± 0.85	2.86 ± 0.99	< 0.001
Number of people living in the same house	4.60 ± 0.73	5.45 ± 1.03	< 0.001
Number of people sleeping in the same room with the child	2.68 ± 1.02	3.27 ± 0.48	< 0.001
	**Mean ± SD**	**Mean ± SD**	**p-value**
Vitamin_D	37.0 ± 10.1	33.3 ± 14.0	0.065
Zn	0.73 ± 0.15	0.70 ± 0.13	0.187
Cu	1.22 ± 0.29	1.30 ± 0.28	0.065
Cu/Zn	1.73 ± 0.50	1.89 ± 0.52	0.060
IgE	51.30 ± 20.98	53.14 ± 18.59	0.574
Eosinophil	3 ± 1	3 ± 2	0.133
	**Control**	**Wheezy**	
**Variable**	**n (%)**	**n (%)**	**p-value**
Gender, male	43 (48.3%)	46 (51.7%)	0.480
Consanguineous marriage	39 (57.4%)	29 (42.6%)	0.134
Prenatal smoking (+)	5 (20%)	20 (80%)	0.001
Postnatal smoking (+)	19 (38.8%)	30 (61.2%)	0.042
Attends daycare	7 (23.3%)	23 (76.7%)	0.001
Poor economical status	21 (47.7%)	23 (52.3%)	0.641
Use of stove for heating	17 (35.4%)	31 (64.6%)	0.10
Number of people living in the same house >5	20 (69.0%)	9 (31.0%)	0.028
Person to bedroom ratio >1	13 (54.2%)	11 (45.8%)	0.709
Breastfed >6 months	38 (53.5%)	33 (46.5%)	0.506
Vit. D supplementation	63 (49.6%)	64 (50.4%)	0.522
Sufficient dietary Vit. D	63 (50.4%)	62 (49.6%)	0.876
TE supplementation	12 (48.0%)	13 (52.0%)	0.769
Sufficient dietary TE	46 (57.5%)	34 (42.5%)	0.072

In control group of our study, the serum level of VD was 26.07 +/- 13.92, Zn was 0.62 +/- 0.1, Cu was 1.49 +/- 0.29 and Cu/Zn ratio was 2.33 +/- 0.48 in infants with history of passive smoking while the serum level of VD was 34.59 +/- 7.29, Zn was 1.83 +/- 0.5, Cu was 1.15 +/- 0.27 and Cu/Zn ratio was 1.83 +/- 0.5 in infants without history of passive smoking. The difference between vitamin D and Zn levels of these two groups were not statistically significant (p = 0.231 and p = 0.630, respectively), whereas the difference between Cu and Cu/Zn ratio were found to be statistically significant (p = 0.006 and p = 0.015, respectively). In study group, the serum level of VD was 34.59 +/- 7.29, Zn was 0.66 +/- 0.75, Cu was 1.44 +/- 0.25 and Cu/Zn ratio was 2.25 +/- 0.44 in infants with history of passive smoking while the serum level of VD was 37.83 +/- 10.79, Zn was 0.66 +/- 0.15, Cu was 1.44 +/- 0.25 and Cu/Zn ratio was 2.25 +/- 0.44 in infants without history of passive smoking. Serum vitamin D levels did not show any statistically significant difference between these two groups (p = 0.231); but serum Zn, Cu, Cu/Zn ratio were significantly different (p = 0.015, p < 0.000, p < 0.000, respectively).

Vitamin D level of healthy infants (only breastfed) was 38.28 +/- 10.26; whereas it was 36.74 +/- 10.11 in the other group. This difference was not statistically significant (p = 0.819). Vitamin D level of infants (only breastfed) with wheezing was 26.18 +/- 13; whereas it was 29.27 +/- 12.26 in the other group. This difference was not statistically significant (p = 0.495).

Thirty-two of seventy-three children (43.8%) had more than three wheezing attacks (recurrent wheezing). The API index was positive in 26 patients (35.6%). When classified to temporal pattern of wheeze, fifty-three of study group (72.6%) were episodic wheezing and the remainder (27.4%) was multiple-trigger wheezing. The mean age of onset of symptoms was 3.58 ± 3.02 months.

We found no significant difference between the study and control group in terms of vitamin D and trace elements. The VD and Zn levels were significantly lower and serum Cu and Cu/Zn ratio were significantly higher in patients with recurrent wheezing, positive API and MTW phenotype compared with patients with non-RW, negative API and EW phenotype (Table 2).

**Table 2:** **Serum levels of vitamin D, zinc, copper and zinc/copper ratio in the study population**

	Control	Wheezy		nRW	RW		API-	API+		EW	MTW	
	Mean ± SD	Mean ± SD	p	Mean ± SD	Mean ± SD	p	Mean ± SD	Mean ± SD	p	Mean ± SD	Mean ± SD	p
Vitamin D	37.0 ± 10.1	33.3 ± 14.0	0.065	37.5 ± 13.8	27.8 ± 12.5	0.03	37.3 ± 13.0	26.0 ± 13.0	0.001	35.3 ± 14.0	27.8 ± 12.7	0.039
Zn	0.73 ± 0.15	0.70 ± 0.13	0.187	0.76 ± 0.11	0.63 ± 0.10	<0.001	0.75 ± 0.12	0.62 ± 0.10	<0.001	0.72 ± 0.13	0.64 ± 0.10	0.019
Cu	1.22 ± 0.29	1.30 ± 0.28	0.065	1.23 ± 0.22	1.39 ± 0.32	0.013	1.20 ± 0.23	1.49 ± 0.26	<0.001	1.23 ± 0.23	1.49 ± 0.31	<0.001
Cu/Zn	1.73 ± 0.50	1.89 ± 0.52	0.060	1.66 ± 0.37	2.19 ± 0.53	<0.001	1.63 ± 0.36	2.35 ± 0.44	<0.001	1.74 ± 0.41	2.29 ± 0.57	<0.001

Viral positivity was 79.5% in study group. The subtype of viral positivity was as following: RSV was 56.2%, rhinovirus was 13.7%, and others were 9.6%. The VD and Zn were significantly lower and Cu/Zn ratio was significantly higher in patients with positive viral serology. The VD and Zn were significantly lower in patients with positive respiratory syncytial virüs (RSV). The Zn level was significantly lower in patients with rhinovirus. The level of Cu and Cu/Zn ratio was significantly higher in patients with rhinovirus (Table 3).

**Table 3:** **Levels of serum vitamin D, zinc, copper and Zn/Cu ratio, according to viral serology of the study population**

	Viral serology (-)	Viral serology (+)	p	RSV (-)	RSV (+)	p	Rhinovirus (-)	Rhinovirus (+)	p
	Mean ± SD	Mean ± SD		Mean ± SD	Mean ± SD		Mean ± SD	Mean ± SD	
Vitamin D	50.33 ± 10.58	28.87 ± 11.16	<0.001	37.5 ± 16.44	29.98 ± 10.91	0.022	34.45 ± 13.96	25.87 ± 12.70	0.072
Zn	0.82 ± 0.13	0.67 ± 0.10	<0.001	0.73 ± 0.15	0.67 ± 0.01	0.036	0.71 ± 0.12	0.60 ± 0.10	0.010
Cu	1.24 ± 0.25	1.32 ± 0.28	0.327	1.37 ± 0.31	1.25 ± 0.24	0.068	1.28 ± 0.27	1.46 ± 0.29	0.047
Cu/Zn	1.53 ± 0.34	1.98 ± 0.52	0.002	1.86 ± 0.53	1.90 ± 0.51	0.739	1.83 ± 0.5	2.22 ± 0.51	0.031

We also tried to determine the relationship between serum level of VD, Zn, Cu and Cu/Zn ratio with severity of disease and use of the healthcare system. The VD and Zn levels were significantly lower and Cu and Cu/Zn ratio were significantly higher in patients with severe wheezing attacks, hospitalized or internalized to intensive care units (ICU) groups rather than mild attack, followed up in outpatient clinic, or internalized to relevant department. Also, we found a positive correlation between CBSS and length of stay (LOS) with Cu and Cu/Zn ratio in this group and negative correlation with VD and Zn. There was also a positive correlation between the number of RTSs in the previous year and thus admission to the emergency room (ER) and hospitalization with Cu and Cu/Zn ratio while there was a negative correlation with VD and Zn in the study group (Table 4).

**Table 4:** **Vitamin D, Zn, Cu levels and Cu/Zn ratio according to severity of wheezing**

	Severe	Non-severe	p	Inpatients	Outpatients	p	PICU	Ward	p
	n:	n:		n:	n:		n:	n:	
Vitamin D	27.17 ± 9.9	35.43 ± 14.69	0.026	31.23 ± 13.81	41.073 ± 12.38	0.015	24.84 ± 10.73	35.28 ± 10.04	0.011
Zn	0.63 ± 0.10	0.72 ± 0.12	0.007	0.68 ± 0.12	0.79 ± 0.11	0.002	0.63 ± 0.11	0.73 ± 0.12	0.019
Cu	1.46 ± 0.33	1.25 ± 0.24	0.003	1.33 ± 0.28	1.17 ± 0.21	0.035	1.51 ± 0.34	1.25 ± 0.24	0.001
Cu/Zn	2.28 ± 0.59	1.75 ± 0.42	<0.001	1.99 ± 0.52	1.49 ± 0.25	0.001	2.35 ± 0.56	1.78 ± 0.45	<0.001

We found a significant negative correlation between VD and Zn levels with the number of RTIs and wheezing attacks and thus admission to ER and hospitalization in the previous year, the severity of attack and length of stay in hospital whereas there was a positive correlation between Cu and Cu/Zn ratio with the number of RTIs and wheezing attacks and the severity of attack (Table 5).

**Table 5:** **The results of bivariate correlations between number of wheeze attacks and serum levels of vitamin D, Zn, Cu and Cu/Zn ratio in wheezy group**

	Number of Wheezing Episodes r*	p-value	CBSS r*	p-value	LOS r*	p-value	RTIs r*	p-value	Number of Emergency Department Visits r*	p-value	Hospitalization r*	p-value
Vitamin D	-0.394	0.010	-0.365	0.002	-0.436	<0.001	-0.286	0.014	-0.307	0.008	-0.406	<0.001
Zn	-0.408	<0.001	-0.511	<0.001	-0.520	<0.001	-0.439	<0.001	-0.394	0.001	-0.484	<0.001
Cu	0.428	<0.001	0.297	0.011	0.338	0.003	0.274	0.019	0.345	0.003	0.411	<0.001
Cu/Zn	0.507	<0.001	0.499	<0.001	0.538	<0.001	0.455	<0.001	0.455	<0.001	0.556	<0.001

*CBSS* Bronchiolitis clinical score, *LOS* Length of hospital stay, *RTIs* Respiratory tract Infections.

*Spearman’s rank correlation test.

## Discussion

In this study, we aimed to show the relationship between wheezing and trace elements and vitamin D. To the best of our knowledge, this study is the first trial to study both vitamin D and trace elements in wheezy infants. We found no overall significant difference between the study group and controls in terms of serum level of Zn, Cu and Vitamin D. Regarding our first and second aim, we found significant relationship between recurrence and temporal pattern of wheeze with serum level of VD and TE. In the RW group, positive API and MTW, serum VD and Zn were significantly lower and serum Cu and Cu/Zn were significantly higher than non-RW, negative API and EW group. With these findings, we can suggest that serum level of Zn, Cu and VD might have role in pathogenesis, recurrence, severity and temporal pattern of wheeze and implicitly, in the pathogenesis of asthma.

Kappele et al. reported that a strong positivity of API, phenotype of MTW and severe attack might be a risk for developing of asthma [11]. It has also been reported that deficiency of vitamin D and Zn can be related to RTIs and this situation can increase the incidence of wheezing and cause asthma attacks to be intensified [18]. In some studies, there were no significant differences between children with RW and healthy children in terms of serum vitamin D and this finding was attributed to small study samples [19]. In several trials, the serum level of Zn was significantly lower in children with RW compared with healthy children and it has been postulated that this lower level of Zn might be a risk factor for wheezing in early childhood [20–23].

It has been shown that the serum level of VD was significantly lower [24], and several other studies have reported that the serum level of Zn was significantly lower [25]. However, the Cu and Cu/Zn ratio was significantly higher in patients with asthma compared with a healthy population [26]. Other trials have failed to show any differences between children with asthma and healthy children in terms of Cu and Zn [27, 28].

The increase of Cu/Zn ratio can be occasionally seen in inflammatory disease in which free oxygen radicals are effective in the pathogenesis of disease. It has been postulated that the increase of serum Cu might cause diminishment of serum Zn and thus it can implicitly cause inflammation by decreasing the capacity of the antioxidant system [29]. Schwarts and Weiss have shown the negative correlation between wheezing and serum Zn/Cu ratio (the inverse ratio of Cu/Zn) [30]. The serum level of Zn was significantly lower and serum Cu and Cu/Zn ratio were significantly higher in asthma patients and this was attributed to the increase of serum Cu [26, 28, 29]. Although the level of serum Cu and Zn were controversial in patients with RW and asthma, the increase of Cu/Zn ratio was thought to be more important than their separate increases or reductions of levels and thus might have role in course of wheezing.

The free radicals developed from ROS can be responsible for pathogenesis of many diseases due to their dangerous effect on cells and tissues. There are some antioxidant defense systems such as mitochondrial cytochrome oxidase, superoxide dismutase (SOD), and glutathione peroxidase (GSH-Px) in the body to prevent the harmful effect of free radicals.

In the literature, the studies about hair Zn levels of tobacco smokers have contradictory outcomes [31, 32]. Kocyigit et al. reported that plasma copper concentration and erythrocyte Cu-Zn SOD activity were significantly higher in tobacco smokers [33]. These findings suggest that antioxidative enzyme activities change depending on their cofactor concentrations in tobacco smokers [33]. In our study, vitamin D levels and smoking did not show any correlation. Similarly, Banihosseini et al. found no relationship between smoking and vitamin D levels of infants and their mothers [34].

These defense systems reserve some trace elements like Zn and Cu. The deficiency of vitamin D is about 25% in Turkey [35] as worldwide [36]. The deficiency of Zn is approximately 9.5% in developed countries and 33.5% in developing countries [37].

In Turkey, infants take 400 IU of Vitamin D daily until the 12^th^ month as in Europe. It is administered by the Ministry of Health without any cost to the families. In this study, we found decreased serum vitamin D levels in infants with wheezing, compared to healthy controls. There is a possibility that this difference might be due to the prolonged indoor stay of the infants with wheezing.

The lower serum level of Zn was also shown in our country [38]. The decreased intake of antioxidant might be related to increased risk of RW and asthma due to a simultaneous deficiency of trace elements [15].

It has also been revealed that there is a decrease in SOD and GSH-Px activity in the nasopharynx aspirate of children with severe RSV bronchiolitis [39]. The influenza and rhinovirus infections can induce the ROS production [40]. The increased ROS in lower RTIs can cause hypersensitivity by deranging the respiratory tract epitels [14].

In our study, the serum level of Zn and VD was significantly lower in RSV positive cases, and the level of Cu and Cu/Zn ratio were significantly higher in rhinovirus positive cases. The children with wheezing who had lower serum levels of Zn and VD, and higher levels of Cu and Cu/Zn ratio suffered from more RTIs and severe wheezing attacks in the previous year, and thus were excessively admitted to the ER, stayed in hospital longer, and necessitated more ICU settings. Various studies have shown a relationship between lower VD levels and hospitalization [41, 42]. However, a study from Canada failed to show this relationship [43]. In another study with RW patients, it has been determined a negative correlation between the serum level of Zn and the number of RTI and wheezing attacks [19]. It has also been reported that there is an association between VD deficiency and the number of asthma attacks in children with asthma [24].

This study revealed the relationship between RW and vitamin D and trace elements. The risk of having recurrent wheezing after acute bronchiolitis might be elucidated with the findings of this study. The ensuing risk of asthma after RW might also be estimated with some other findings of our preliminary study in this area. The discussion about adding the VD or trace elements in foods or formulas or giving separately as a drug becomes more of an issue. This trial might provide preliminary data for hypothesis postulating reduction of wheezing upon vitamin D and zinc supplementation.

The roles of VD and TE in RW are still controversial and their effect on immune system should be discussed by future studies. How VD and TE can effect therapeutically in treatment of wheezing should require larger and controlled trials. The screening of serum level of VD and TE on admission of AB can be useful in estimating the risk of being RW.

There are some limitations in this study. First of all, the sample size is relatively small, which decreases the power of the study. Statistically insignificant associations might become significant in future studies with larger sample sizes. Since our study is a cross-sectional design, the accurate cause-effect relationship cannot be formed, and the response of families may not be correctly related to our results.

Further studies are necessary to show the relationship between wheezing and trace elements for the prevention of disease and improvement of clinical course.

## Conclusions

It may be postulated that for the determination of asthma risk in patients with recurrent wheezing, the serum level of vitamin D, copper and zinc can be used as a routine biomarker beside the API index and temporal pattern of wheeze, and used in the clinical course and follow-up of patients. This can also be useful for preventive medicine. The supplementation of vitamin D and zinc for children who are lack of them might be practical and favorable for helping the better control of wheezy children.

## Abbreviations

API: Asthma predictive index
ERS: European respiratory society
EW: Episodic wheeze
MTW: Multiple-trigger wheeze
VD: Vitamin D
TE: Trace elements
RW: Recurrent wheezing
ROS: Reactive oxygen species
Zn: Zinc
Cu: Copper
RTIs: Respiratory tract infections
RR: Respiratory rate
CBSS: Clinical bronchiolitis severity score
RSV: Respiratory syncytial virus
ICU: Intensive care unit
LOS: Length of stay
ER: Emergency room
SOD: Superoxide dismutase
GSH-Px: Glutathione peroxidase.
